# A Decentralized Peer-to-Peer Remote Health Monitoring System

**DOI:** 10.3390/s20061656

**Published:** 2020-03-16

**Authors:** Muhammad Salek Ali, Massimo Vecchio, Guntur D. Putra, Salil S. Kanhere, Fabio Antonelli

**Affiliations:** 1 OpenIoT Research Unit, ICT Center, Fondazione Bruno Kessler, 38100 Trento, Italy; ms.ali@fbk.eu (M.S.A.); fantonelli@fbk.eu (F.A.); 2 School of Computer Science and Engineering, University of New South Wales, Sydney NSW 2052, Australia; gdputra@unsw.edu.au (G.D.P.); salil.kanhere@unsw.edu.au (S.S.K.)

**Keywords:** blockchains, IoT, healthcare, remote health monitoring, privacy, trust, trustless architectures

## Abstract

Within the Internet of Things (IoT) and blockchain research, there is a growing interest in decentralizing health monitoring systems, to provide improved privacy to patients, without relying on trusted third parties for handling patients’ sensitive health data. With public blockchain deployments being severely limited in their scalability, and inherently having latency in transaction processing, there is room for researching and developing new techniques to leverage the security features of blockchains within healthcare applications. This paper presents a solution for patients to share their biomedical data with their doctors without their data being handled by trusted third party entities. The solution is built on the Ethereum blockchain as a medium for negotiating and record-keeping, along with Tor for delivering data from patients to doctors. To highlight the applicability of the solution in various health monitoring scenarios, we have considered three use-cases, namely cardiac monitoring, sleep apnoea testing, and EEG following epileptic seizures. Following the discussion about the use cases, the paper outlines a security analysis performed on the proposed solution, based on multiple attack scenarios. Finally, the paper presents and discusses a performance evaluation in terms of data delivery time in comparison to existing centralized and decentralized solutions.

## 1. Introduction

In 2008, blockchains gained popularity among distributed ledger technologies when the Bitcoin whitepaper was published [1]. Within the Bitcoin application, the utility of blockchain is to facilitate the exchange of digital tokens among participants in a peer-to-peer network, without the need to place trust in a centralized third party entity. Such transactions have traditionally relied on banks and other mediating third party services. Involving third party entities for mediating transactions is less than ideal for a number of reasons: the trusted third party may malfunction, it may be compromised by malicious actors to render the system unsecure. The motivation behind blockchains, and indeed digital ledger technologies, is to eliminate the reliance on trusted third parties to perform transactions and exchanges with accountability. Blockchains are decentralized ledgers that hold records of transactions or data exchanges that take place in a peer-to-peer network. The ledger is replicated over the peer nodes, and a canonical shared state of the ledger is maintained through algorithmic consensus among the blockchain network peers. Circumventing the reliance on centralized authorities, as well as the immutability and security of its contents, is the reason for the research popularity that blockchain has received in recent years.

Building on the implementations of blockchain within the cryptocurrency space, the third-generation of blockchain technology (blockchain 3.0) aims to branch out into non-financial applications [2], most notably so within the Internet of Things (IoT) [3]. The IoT has gone through exponential growth and is termed a revolution to modern life and industry [4]. The IoT within itself is a network of interconnected devices that automates tasks and services. The edge of the IoT, closer to the consumers, contains sensor devices that collect data from their surroundings, and, through these data, IoT platforms provide services to aid our modern lives. Despite the benefits and convenience of the IoT, the sheer volume of data collected and managed by centralized entities raises privacy concerns [5]. Operating on a centralized client-server access model, IoT service provision exposes IoT users’ personal data to third party entities, which routinely glean through it to ascertain users’ behaviours and preferences, to benefit from them in targeted advertisement campaigns [6]. The need of the hour is to develop systems that empower users’ privacy and enable data ownership for IoT users.

A decentralization of the IoT thus becomes a necessity of the modern age, in order to maintain individual liberty and the convenience of an interconnected world. Researchers have turned their attention towards blockchain as a tool to decentralize the IoT, and eliminate the necessity of heavily centralized architectures [3]. The inherent mechanisms involved in blockchain networks enable decentralized data exchange, authentication and authorization without the need for a centralized intermediary. However, the challenges associated with a blockchain-IoT convergence are related to privacy and scalability [3,7]. Blockchain contents, being replicated over all peer nodes, may expose any sensitive data shared over the blockchain. Furthermore, since there is algorithmic consensus involved to maintain a canonical shared state, there is an inherent latency and a limit to the transaction throughput that blockchains can provide. A more practical and feasible solution would therefore be a hybrid of *“on-chain”* and *“off-chain”* solutions, where authentication and record-keeping duties are kept the responsibility of the blockchain, while other non-blockchain technologies can shoulder the burden of data transfer.

With the growing research interest in integrating blockchains into the IoT, healthcare is one of the most significant industries where a number of suitable use-cases have been identified. These include applications in medical record keeping, pharmaceutical supply chains, health insurance, health research analytics and remote health monitoring [8]. Remote health monitoring (RHM) enables doctors to monitor patients’ physiological conditions from their homes, while freeing up expensive healthcare facilities and hospitals to those in urgent need. For the patients, RHM provides a more comfortable and cost-effective alternative to on-site monitoring. Non-invasive wearable sensor technologies are proven to be viable diagnostic tools for monitoring patients’ physiological signs and activities remotely, in real time [9]. Naturally, wearable sensors and the data generated by them to facilitate RHM have been the subject of research and development in RHM systems.

The contribution of this paper is a decentralized solution which aims to provide scalable and private-by-design remote health monitoring. Leveraging blockchain’s capabilities in decentralized accountability, authorization and authentication, our proposed solution is designed to securely share patients’ sensitive data with doctors, without having the data pass through third party services.

To demonstrate and analyze the proposed solutions’s application in real-world RHM scenarios, we developed a real-world implementation, and explored three RHM use cases: cardiac patient monitoring, sleep apnoea testing and EEG monitoring for epilepsy. Our implementation is built on Ethereum’s Rinkeby test network, which provided an adequate platform for testing our solution’s applicability in a blockchain network spread worldwide. For delivering patients’ health monitoring data, we have implemented Tor hidden services. We chose Tor instead of other peer-to-peer data delivery solutions due to the fact that through Tor; we are able to transfer the data without any unnecessary metadata which is not relevant to the application.

The remainder of this paper is organized as follows: Section 2 outlines the architecture and components of our proposed RHM solution. Section 3 delves into the design specifics and working principles of the proposed solution over three RHM use-cases. Section 4 contains a security analysis which addresses threats that the solution may face, and how it mitigates them. Section 5 details a performance analysis we carried out on the solution, in comparison to existing centralized and decentralized solutions. In Section 6, we discuss previous related work in developing blockchain-based solutions for remote health monitoring. Finally, in Section 7, we summarise our findings and conclude the paper.

## 2. The Proposed Decentralized RHM System

Building a blockchain-based solution involves tackling a four-way trade-off between decentralization, scalability, privacy and latency [3]. While public blockchains offer great accountability and censorship resistance, blockchains may not be suitable in use-cases where streams of data need to be sent from patients to doctors. The transaction contents are openly accessible to all members of the blockchain network, which can impede patients’ privacy. Even in cases where the contents of transactions are encrypted, the transaction throughput provided by blockchains is not high enough for a near-real time data transfer. To complicate things further, there is a scalability/throughput trade-off between public and private blockchain deployments. While private blockchains provide higher transaction throughput, the voting mechanisms involved in private blockchain deployments severely limit scalability.

In general, our proposed RHM solution is built on an architecture consisting of a public blockchain for accountability, and Tor hidden services to relay patient data directly to doctors. Our blockchain-based system is utilized on-chain techniques primarily for privacy-preserving identity management, agreements and record-keeping, in a secure and immutable way. Conversely, our proposed architecture is using off-chain mechanisms, i.e., Tor hidden services, for data delivery to maintain reasonable latency. Together, the on-chain and off-chain mechanisms form a decentralized, accountable, secure and private-by-design framework for health monitoring. Please note that our private-by-design framework also helps to provide an alternative to patients who are afraid of healthcare data breach, which has proven to be a serious concern over the recent years [10].

In this section, we outline the decentralized technologies used in developing our proposed solution, as well as the network architecture designed to mitigate the privacy and scalability concerns that arise when decentralizing remote health monitoring. Blockchains, along with decentralized peer-to-peer data transfer mechanisms, form the basis of a secure and accountable health monitoring system, without relying on third party servers.

### 2.1. Core Components of the RHM System

#### 2.1.1. Blockchain

Blockchains are distributed time-stamped records of all network transactions, replicated over the nodes of a peer-to-peer network. Block validator nodes participate in consensus algorithms, for validating and appending new blocks to the blockchain, as well as maintaining an immutable, canonical shared-state of the blockchain. Transaction information is grouped together into blocks, and each block is linked to its predecessor block, similar to a linked list. In order to perform a modification attack, an adversary would have to alter the contents of a single block, as well as all the subsequent blocks, over a majority of the blockchain peers at the same time. Therefore, to further the degree of decentralization, and for robustness and security in blockchain deployments, it is necessary to have a large block-validator pool [3].

For the proposed framework, the basic components required are the blockchain itself, smart contracts for programmable service-level agreements, and decentralized file storage for hosting the application data. Further pertinent details on blockchains, and the specific consensus algorithms used for the proposed framework are discussed below:

- **Transactions and Addressing:** Each peer $y_{r}$ on the blockchain has a public/private keypair $\left\{k_{p}^{r},k_{s}^{r}\right\}$ which is used for addressing, and creating digital signatures on each transaction, for guaranteed non-repudiation. Since these keypairs are not associated with real-life identities, blockchains offer *“pseudonymity”* to its users [7]. Signed transactions are made for transferring cryptocurrency tokens, or interacting with the Application Binary Interface (ABI) of functions written in deployed smart contracts.
- **Smart Contracts:** A smart contract is simply a piece of code stored in the blockchain itself and able to enforce programmatic terms and conditions over transactions occurring in the network. In our proposed framework, for private-by-design IoT data transactioning, we use smart contracts to enable IoT users to decide when and how much data to share with entities of their choosing, in exchange for monetary incentives and/or services.
- **Consensus Algorithm:** The most commonly used consensus algorithm for publicly deployed blockchains is proof-of-work (PoW), commonly known as ‘mining’ [1]. Miner nodes group new transactions into blocks, and engage in a race to find a nonce that, when hashed with the candidate block, produces a resultant which satisfies a predetermined difficulty level. The nonce serves as a verifiable proof of computational work done. The proof-of-work consensus algorithm provides fault tolerance of n≥2f+1, where *n* is the total computational power of the network, and *f* is the computational power of an adversary to the network. In public blockchains, the throughput can be evaluated using the parameters of block-size and block-interval, where block-interval is the average time required to publish a new block [11]. The transaction throughput can thus be theoretically calculated by:
  (1) $$\Omega \left(S_{b},T_{i}\right)=\frac{\left[S_{b}\;/\;\chi \right]}{T_{i}}$$
  where $S_{b}$ represents block-size, $T_{i}$ represents the average block-interval, and χ is the average transaction size.
  For our solution, we used proof-of-authority (PoA), a variation of PoW, which limits mining only to delegated block validators. In this consensus mechanism, block validation is not fully decentralized and open to the public; however, with a sufficiently large validator pool, the immutability of the blockchain contents will remain secure. In a national healthcare use-case, the validator pool would consist of a consortium of hospitals and healthcare providers. An alternate to this is the voting-based Practical Byzantine Fault Tolerance (PBFT) consensus algorithm [12]. Under PBFT, new candidate blocks are validated through multiple rounds of voting among block validators, instead of expending computational effort. PBFT is computationally efficient, with a fault tolerance of n≥3f+1, where *n* is the total number of validating nodes, and *f* is the number of compromised or faulty nodes. While PBFT is computationally effiicient, it is only suitable for private deployments, since the high network overhead severely limits the scalability of the validator pool.

For applications involving data transfer, relying on a blockchain alone would lead to privacy issues and exponentially increasing storage requirements [2]. Therefore, for accountability and decentralized messaging systems, off-chain data transfer mechanisms are an important addition to the decentralized application stack. One such example is the peer-to-peer BitTorrent protocol [13]. With blockchain-based accountability and identity management, BitTorrent does allow for decentralized healthcare data transfer; however, it does not have sufficient privacy guarantees. Privacy guarantees require that any data that are not of primary relevance to the healthcare application should not be exposed. BitTorrent traffic can leak users’ IP addresses, location information and other metadata [14]. On the other hand, with adequate design considerations, Tor masks any metadata associated with the patients that intend to send their doctors any real time monitoring data. We have used the Ricochet protocol in our application for end-to-end encryption, to ensure that patients’ sensitive data are not exposed at the final relay node of the Tor network.

#### 2.1.2. Tor Network

Tor is a decentralized, privacy enhancing system designed to prevent traffic analysis attacks [15]. Tor provides a layer of privacy protection on top of TCP, while maintaining high throughput and low latency, which makes it ideal for data transfer applications. Since its initial release, researchers have conducted analyses on Tor’s performance [16] and security [17].

Tor provides a layer of privacy protection for TCP through a three-hop routing path, using a layered encryption strategy as seen in onion routing mechanisms. Additional considerations for end-to-end encryption are required since the entrance Tor router can directly observe the originator of a particular request through the Tor network, and the exit node can examine the unencrypted payload, and the destination server. No single node can observe the sender and receiver. To achieve low latency, Tor does not re-order packets within the Tor network.

Ricochet (https://ricochet.im/) is an anonymous peer-to-peer instant messaging system that operates on Tor hidden services, which is used to relay messages without relying on centralized messaging servers. Being run on Tor hidden services, Tor follows rendezvous specifications [18] with self-authenticating hostnames. When establishing a Tor hidden service, a 1024-bit RSA key pair is generated, and a SHA-1 digest of the public key is calculated. The .onion address is then the base32-encoded first half of the SHA-1 digest. To be noted is the fact that the RSA keypair is what maintains secure transmission, and SHA-1 is only used to prepare the .onion address from the RSA keypair.

Users would be able to access this hidden service through the .onion address, for example: *2qxw7mf2xnfh4mqr.onion*. In Ricochet, the contact ID follows the following format: *ricochet: 2qxw7mf2xnfh4mqr*.

### 2.2. Architecture of the RHM System

Blockchains, from a data structure perspective, are distributed databases with decentralized consensus on the entries being added to them. In applications involving data transfer, a blockchain can be used to create an immutable log of all data generation and access events, while smart contracts can be used to program responses to certain events or to enforce service level agreements [19]. Subsequently, this log can be used for transparency and accountability.

However, this poses unique challenges in the IoT domain. First, since all records in publicly deployed blockchains can be read by every node in the network, issues related to privacy arise. While blockchain addressing does provide pseudonymity, inferences can be made about accounts that generate specific kinds of data. In the IoT, prevalent privacy issues pertain to third-party entities monitoring user behaviour and preferences, and logging all IoT data generation events on a public blockchain will lead to privacy breaches [3]. Furthermore, for smart healthcare applications, maintaining healthcare records and transferring monitoring data over a single publicly deployed blockchain severely limits scalability. In our scenario, an RHM system measures and transmits patient’s data in textual format of approximately 1 to 2 kilobytes per second per patient. Considering the high frequency and volume of data generated in health monitoring applications on a nationwide scale, the added latency of the consensus algorithm will be a bottleneck to record every data transfer event. Other than this, it is worth recalling that blockchains are linearly growing data structures. Therefore, to log every single data transfer event on a public blockchain will cause an explosion in the storage requirements of blockchain full-nodes.

**Figure 1:**
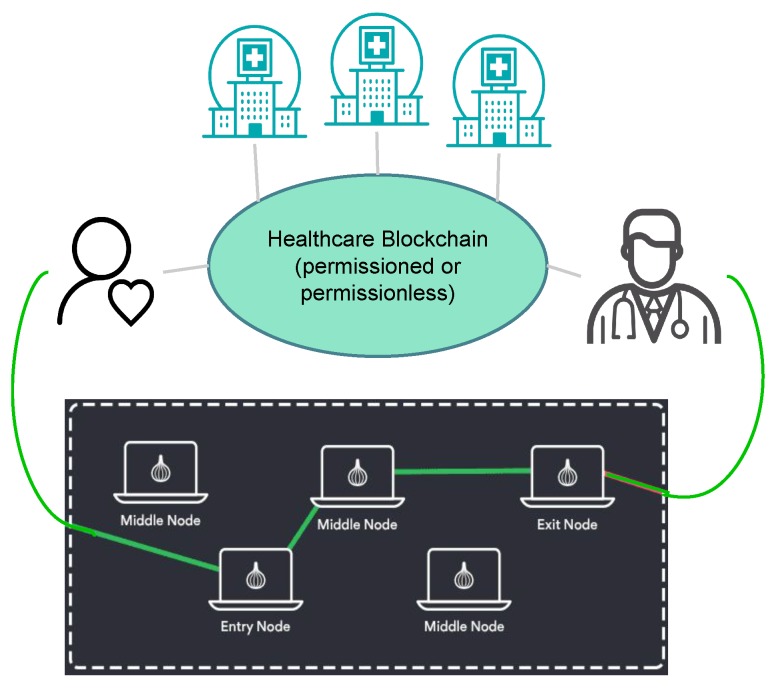
The proposed blockchain-based remote health monitoring (RHM) architecture.

To address these limitations, we have implemented an architecture consisting of a public blockchain at the core of the health monitoring system, with Tor hidden services connecting patients to their doctors, as illustrated in Figure 1. This architecture allows patients to register themselves with a particular healthcare provider, and engage in agreements about the nature of the data shared directly with the healthcare professional. The healthcare blockchain stands to provide accountability, identity management and a medium for maintaining healthcare registration information for patients. None of the patients’ actual health monitoring data are stored on the blockchain, and the agreements on the nature of the data being shared are kept encrypted. In the event of an investigation or an insurance claim, the healthcare provider can present details of the agreements between the patient and the doctor, while the patient’s monitoring data remains private.

The public healthcare blockchain consists of hospitals, and doctors who are registered with specific hospitals. While registering with a specific doctor, the patient and doctor both exchange their .onion addresses, which are kept encrypted in the blockchain’s transaction payloads. The patient transmits their health monitoring data to the doctor via Ricochet, and logs the starting and stopping timestamp onto the public blockchain, along with the overall size of data transmitted during that time. These logs provide accountable proof of the patient having transmitted their healthcare data in accordance with the doctor’s request. The doctor’s request for data and subsequent recommendations, all encrypted, provide evidence of the medical advice given to the patient.

#### Privacy and Scalability Benefits

Using off-chain data transfer mechanisms, and using blockchains simply for negotiations and record-keeping in remote health greatly reduces the load on the blockchain system. With the overall number of interactions with the blockchain greatly reduced on a per-patient basis, the health monitoring blockchain system can scale up to a nationwide level. Furthermore, with advancements in “*sharding*” [20] on the Ethereum platform, it will become possible to scale up the health-monitoring blockchain nationwide, with different shards being accountable for different regions. Using hidden Tor services and the Ricochet protocol greatly adds to the privacy that is built into the proposed solution. None of the patients’ health data are accessible to anyone in the health-monitoring system except the doctor it is intended to be shared with. With end-to-end encryption, the patients’ health data are not accessible to anyone over the Tor network. The solution does not involve third party servers in relaying patient data, and none of the data are stored onto the blockchain. By using Tor hidden services, we ensure that no metadata are transmitted to the other side which is not primary to the application itself.

## 3. Remote Health Monitoring Use-Cases

The proposed blockchain-based remote health-monitoring solution provides a medium for negotiation and accountability, identity management and a private and secure means to transmit health monitoring data. In this section, we will outline the decentralized accountability mechanism involved, as well as the working principles of the patient–doctor relationship under the proposed solution. We have looked at three main use-case scenarios where the solution can be applicable, out of many more. Firstly, the cardiac patients who require longer term monitoring; secondly, sleep apnoea studies that require short-term monitoring over specific periods of time, and thirdly epileptic patients who may need to transmit EEG data in the event of a seizure, on an emergency basis.

**Figure 2:**
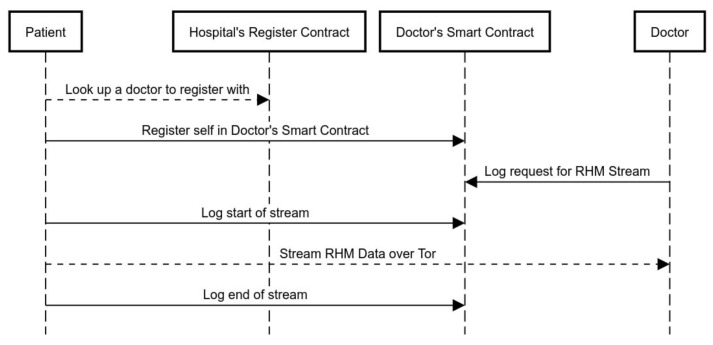
Sequence diagram of a remote health-monitoring instance, where a patient registers with the doctor and avails remote health-monitoring services. All interactions with the smart contract represent transactions. Dashed arrows represent off-chain interactions over Tor hidden services.

Firstly, each hospital has a smart contract where doctors “register” themselves with verifiable registration information. “Registration” entails doctors enlisting themselves in a mapping data structure within the hospital’s smart contract, which can be looked up by patients to find the doctor they are to register with. This mapping will include the doctor’s public-facing information, as well as an address to the doctor’s own smart contract, where the patients can register themselves. Figure 2 is an illustration of the step-by-step sequence of events that takes place when a patient registers with a doctor and avails remote health monitoring services.

**Figure 3:**
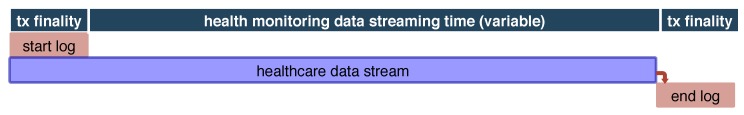
Timeline of health monitoring session, including start and end logs issued to blockchain.

Additionally, Figure 3 shows the timeline of a single remote health monitoring session, from start of session log, to the actual data stream, to the end of session log.

Here, let $H=\{h_{0},h_{1},\ldots ,h_{n}\}$ be the set of hospitals, each being member participants on the remote healthcare blockchain. Additionally, let $Y=\{y_{0},y_{1},\ldots ,y_{n}\}$ be the set of doctors, and $X\;=\;\{x_{0},x_{1},\ldots ,x_{m}\}$ be the set of patients, such that:

(2) $$\forall h_{i}\exists Y^{i}\subset Y:\left(h_{i}∋Y^{i}\right)$$

(3) $$\forall y_{i}\exists X^{i}\subset X:\left(y_{i}∋X^{i}\right)$$

where ∋ signifies registration. While the doctors and patients each have a public-private keypair $\left\{k_{p},k_{s}\right\}$ for issuing transactions on the blockchain, for each doctor–patient pair, there will exist a pair of .onion addresses. When a patient $x_{j}$ invokes the REGISTER() method in the smart contract of a doctor $y_{r}$, the patient encrypts their own .onion address $o_{j}$ with the doctor’s public blockchain key, and sends it to the doctor via the REGISTER() method. Similarly, in return, the doctor’s blockchain node sends over a specific .onion address for the patient:

(4) $$\forall y_{r}\exists \langle k_{p}^{r},k_{s}^{r}\rangle :d_{k_{s}^{r}}\left(e_{k_{p}^{r}}\left(o_{j}\right)\right)=o_{j}$$

(5) $$\forall x_{j}\exists \langle k_{p}^{j},k_{s}^{j}\rangle :d_{k_{s}^{j}}\left(e_{k_{p}^{j}}\left(o_{r}\right)\right)=o_{r}$$

The reason for having two .onion addresses for each doctor–patient relationship is to enable the doctor’s node to end transmission on a specific .onion address if someone transmits data for a longer period of time than what is decided.

With knowledge of the patient’s condition and following off-chain correspondence, a doctor issues a request for data, with parameters based on the urgency or timespan of health-monitoring required. The time period for data transmission is set, the limits for the volume of data to be sent are set, and this time-stamped request logged onto the blockchain serves as an accountable proof of the request.

Following the doctor’s request for data, patients transmit their health-monitoring data over Tor hidden services addressed in Ricochet to the .onion address provided to them by their doctor. The starting time of transmission is logged on to the blockchain, which serves as proof of authentication and time log of the start of data transfer. At the end of the data transfer, the patients log an end of transfer onto the blockchain, which includes a hash of the bulk of data which is sent to the doctor, for verifiability. The time logs will indicate how well a patient has fulfilled the doctor’s request and the immutable contents of the blockchain will provide a hashed proof of the data sent. Following the remote health monitoring, a doctor can provide recommendations, all without having the patients’ data go through a third party server or data management system.

**Cardiac Patients***Long-term RHM:* For cardiac patients, the doctors will issue a request for health monitoring data for a longer period of time. This longer period may include further data transfer parameters like specific times of the day, a specific volume of data during the day, whichever suits the patients’ case best. In this case, the doctor will have to have a specific node listening over the hidden service for a longer period of time. Within the node, at the doctor’s end, there will be safeguards to ensure that no malicious flooding of data are taking place, and only a specified amount of data are being received. In this particular case, real-time analytics can be employed at the patient’s end, with gentle reminders to the patient for being careful during periods of exertion or stress. Predictive models at the doctor’s end can help the doctor modify the course of healthcare being provided to the patient, in a peer-to-peer fashion.

**Sleep Apnoea Patients***Short-term RHM:* For sleep apnoea patients, relevant research efforts have shown that home testing may lead to fewer false-negatives, since patients are sleeping within the comfort of their own beds [21]. For transferring short-term sleep apnoea test data, doctors can request data over a smaller period of time. Here, the specific .onion address on the doctor’s end will cease listening at the end of the specified time period. Such an application of peer-to-peer remote health-care monitoring will allow patients access to doctors beyond their geographical region, and will further the cause of medical globalisation.

**Epileptic Patients***Sporadic RHM:* In the event of an epileptic seizure, patients are rushed to the hospital for a prompt EEG, so doctors can gain insight into the epileptic event. With the improving robustness of home EEG equipment [22], it is possible for doctors to receive data as early as possible following an epileptic seizure. In this case, typically doctors monitor epileptic events that take place over a specified amount of time, and recommend treatment options. The doctor will have a .onion address listening over a period of time, with specified limits of data to be transferred per day. Preventing excessive amounts of data being transferred helps mitigate the network being flooded, and serves the purpose of all genuine patients. Under the proposed solution, patients can transfer near real-time data taken from home EEG equipment, to provide doctors with relevant data in time, without rushing or discomforting the patient.

## 4. Security Analysis

In this section, we outline the security analysis we performed on our proposed blockchain-based remote health monitoring solution based on multiple attack scenarios. We have defined a threat model to highlight the vulnerabilities associated with health monitoring systems, and we have performed a risk assessment to analyse the extent to which the proposed framework addresses the aforementioned vulnerabilities. Though this work focuses on the proposed framework’s design and practical implementation, we have endeavoured to analyse the threats facing the framework, and included our assessment of how risks are mitigated. For a more formalized insight on the threats facing remote health monitoring systems, readers are recommended to read the work by Handler et al. [23]. Additionally, the work [24] contains a security analysis on blockchain technologies in particular.

### 4.1. Threat Model

The threat model defined in this analysis is comprised of assets, threats, attackers and vulnerabilities that can befall a health monitoring system.

#### 4.1.1. Assets

RHM systems have their utility in allowing doctors to observe patients over a decided period of time, while maintaining patients’ comfort. Here, the assets are what the patients have to lose in the event of compromised security.

In the case of successful data modification through a man-in-the-middle attack scenario, healthcare providers may not receive reliable data, which may impede proper health provision, and may even result in loss of life. Metadata surrounding the data transfer being exposed to attackers may reveal information on patients’ location, the severity and nature of their illness, their day-to-day activities, etc. The assets considered in this threat model are as follows, in decreasing order of severity of loss in case of compromised security:

1. Life
2. Health
3. Location
4. Medical privacy
5. Behaviour
6. Activities

#### 4.1.2. Threats

Threats can manifest themselves in either the patients’ data being exposed, modified, or prevented from reaching the healthcare provider. These threats can come from attackers of varied motivations, from inheritors to criminals. Through various attack scenarios, these threats may be aimed to sabotage the healthcare being provided to the patient, or to glean information on patients for nefarious purposes. Out of these threats, the most severe are the threats that modify the patients’ data being transmitted, since false positives and false negatives may hold the greatest impact to the aforementioned assets. Messages being handled through centralized RHM servers have singular points of failure, and may fall victim to insidious attacks that are typical in traditionally centralized IoT solutions [10].

#### 4.1.3. Attacks

We have identified the following attack types, as discussed here. These types of attacks are chosen due to their varying natures and severity in threatening the aforementioned assets within the context of RHM.

1. *Man-in-the-Middle Attacks:* Man-in-the-middle attacks occur when an attacker intercepts communications being transmitted from patients to their doctors, and even gains the opportunity to modify the data being sent. In such an attack, adversaries can send through false data that can potentially prevent timely healthcare provision in highly sensitive cases. Sensitive metadata including location information can be used to monitor patients’ behaviour and identify the patients’ possessions. Computationally capable attackers, such as criminals and burglars, may employ this form of attack to gain insights into the life of a patient.
2. *Denial of Service Attacks (DoS):* Briefly, in a DoS attack, vast amounts of fake requests are sent to a target, in order to overwhelm it and make it unavailable to legitimate users. Such an attack is often performed through a network of infected devices (i.e., a *botnet*) in a distributed manner, hence the name Distributed DoS (DDoS) [25]. If an RHM system falls to a DoS attack, it may have dire consequences. It may put patients at risk and result in care not being provided on time, in case of emergencies. This may be the attack of choice for the less computationally capable attackers, who can use jamming equipment or similar to launch a DoS attack.
3. *Traffic Analysis*: A subset to the man-in-the-middle attack, sniffing and analysing the data traffic being sent may provide insights about the behaviours and location of a patient, thus allowing burglars to anticipate when a patient will be away from home.
4. *Social Engineering*: Attacking the people involved in a system may lead to efficient and harmful attacks to the system. It can be used to gain access to credentials, which can lead to the system being rendered vulnerable to the aforementioned attack vectors.

### 4.2. Risk Assessment

Here, we will discuss how the design of the proposed framework lends itself to mitigating the threats faced by RHM systems, as outlined in the threat model. Our decentralized RHM framework is designed to remove single points of failure and provide improved protection of patients’ privacy, as compared to centralized RHM systems.

#### 4.2.1. Man-in-the-Middle Attacks

In using a decentralized ledger to manage identities and maintain hashes of the data being sent over Tor, the proposed framework performs well to mitigate man-in-the-middle attacks in multiple scenarios, as opposed to traditional centralized RHM systems.

*In the event of deanonymized blockchain addresses:* Using on-chain and off-chain techniques to split the control plane and data plane within the proposed RHM framework has its benefits in protecting the off-chain data streams, even in the event of a patients’ blockchain address becoming deanonymized. The adversary can use a deanonymized blockchain address to view the encrypted negotiation signalling messages being sent over the blockchain. Not only are the contents of the signalling messages kept secret from the adversary, the data being sent, along with the patients’ location data are kept safe in the event of a blockchain address becoming deanonymized.

*In the event of compromised .onion addresses:* For each doctor–patient relationship, the .onion addresses and blockchain keys both serve as multi-factor authentication for engaging in remote health monitoring. The .onion address provided to patients by their doctors comes via a blockchain transaction, which has the doctor’s blockchain signature. On the other end, if a patient’s .onion address is compromised, any data transferred to the doctor will either not have a start of transfer log on the blockchain, or the data hash stored on the blockchain at the end of the transfer will not match the corrupt data that was actually sent.

#### 4.2.2. Denial-of-Service (DoS) Attacks

In the proposed framework, adversaries may aim to launch DoS attacks on the blockchain, or on the .onion service.

*DoS attacks on the the healthcare blockchain:* the healthcare blockchain used by the proposed solution is a distributed system which has a tokenized cost for signing transactions (i.e., transaction fees). All entities involved have only a limited supply of the blockchain’s token, which mitigates the chance of performing a (D)DoS attack on the solution. The hospitals and healthcare companies who maintain the consortium transfer tokens to all entities involved within a limit depending upon multiple factors, for example what insurance a patient has, or whether the entity is a patient or a high ranking oncologist. Moreover, generally speaking, the collective computing power available in the consortium will make it extremely hard to launch successful DoS attacks. The system itself remains safe from a flood of blockchain transactions, and any wrongdoing is easily detectable.

*DoS attacks on the .onion service:* the negotiation stage prior to sending the data involves agreements on the volume of data being sent. In case of a compromised .onion address, an adversary may attempt to flood the data stream. In this case, the data volume agreement is met before the agreed time is up; therefore, wrongdoing is detected and mitigated by exchanging new .onion addresses and resetting the stream.

#### 4.2.3. Traffic Analysis

*Sniffing data sent from a Tor exit relay:* While issuing requests and delivering data through Tor hides, any metadata regarding the location of the source or destination, an issue arises with the last relay node. While the final relay node may not know where the healthcare monitoring data has come from, it can observe the destination, and can make inferences on the sensitive health data. To combat this, we have incorporated Ricochet into our solution to provide end-to-end encryption based on the Ricochet address of the destination entity. This way, none of the data are exposed to any off-chain entity, as well as any entity on the remote healthcare blockchain.

*Sniffing data transfer parameters from the doctor’s smart contract:* Let’s assume an entity on the remote healthcare blockchain $y_{r}^{\prime }$, acting as an adversary, is interested in getting access to the information of the patient $x_{j}$ stored in the smart contract of a doctor $y_{r}$. This information can include the patient’s identity, the volume of data sent to the doctor, as well as the nature of data which was sent. This information, $h_{j}$ is encrypted with $k_{p}^{r}$, thus it can only be decrypted with $k_{s}^{r}$, and with the doctor not having had their account compromised, $y_{r}^{\prime }$ will not be able to get access to the patient’s information.

#### 4.2.4. Social Engineering

*Impersonators and malpractitioners in the hospital’s smart contract:* The hospital maintains a register of doctors within its smart contract, with verifiable information regarding their medical licenses and identification. Any new entries made to the hospital’s register go through a verification stage, where the members of the consortium all verify the medical license of the doctor, and based on a vote, the entry is allowed to be appended to the hospital’s mapping data structure. In the event of malpractice, the hospital simply drops the entry of the doctor in question from its smart contract. The smart contract function used for removing these entries requires digital signatures from the hospital; therefore, no unauthorized member from the blockchain can access this function.

### 4.3. Conclusions

In conclusion, the proposed framework provides a higher degree of protection of both the patients’ privacy and dignity, while not putting their trust on a centralized authority to handle their data. Future work on the design of the framework can still be carried out to further strengthen the framework against malware, blockchain address deanonymisation and blockchain network analysis.

## 5. Performance Analysis

This section details the performance analysis based on the experiments we conducted to validate the proposed solution. The proposed framework was developed and deployed on a Proof-of-Authority (PoA) based blockchain. The solution was implemented on the Ethereum Rinkeby platform, which provides an accurate insight into how well the solution will perform in real-life deployments. As discussed in Section 4, the involved entities all get a limited amount of tokens to engage with the system, which hold no real-world monetary value. We used Rinkeby’s native tokens to visualize the transaction fees incurred when interacting with smart contract functions.

For the performance analysis, we considered the computational overheads on the patient and doctor nodes for participating in the remote healthcare blockchain, as well as the propagation delay involved in delivering health monitoring data over Tor. The most notable aspect of the performance analysis was to compare the performance of our data delivery mechanism against centralized solutions and existing blockchain-based solutions. We used Telegram’s API as a centralized service to compare the data delivery performances. Telegram is a centralized messaging service, which provides an example of the centralized systems current health monitoring systems heavily rely on. For blockchain-based solutions, we used Ethereum’s whisper [26].

Ethereum’s whisper is a communications protocol for distributed applications within the Ethereum blockchain application stack. Whisper claims to achieve ‘darkness’ in that it delivers messages while preventing any metadata to go through. The aim of whisper was anonymity, and, while Whisper does indeed function very well in centralized clusters such as the one used by Status (https://status.im/), it does not succeed in delivering messages over public PoA blockchains. The reason in this is the fact that Ethereum blockchain nodes are not economically incentivized to run the whisper protocol. Thus, in a network such as Rinkeby, you may not have an end-to-end route where all peers in between have whisper enabled. Therefore, for whisper, we have used theoretical values as a comparison.

Messages in whisper are broadcasted over a message bus without traditional “routing”. Within a whisper message’s envelope, some of the parameters required are the TTL, the “PowTime” and “PowTarget”, as seen in the snippet below:

message:= whisperv6.NewMessage{

Payload: []byte("123"),

PublicKey: publicKey,

TTL: 60,

PowTime: 2,

PowTarget: 2.5

}

The last two parameters signify the amount of time given to perform proof-of-work on each message. This is whisper’s way of preventing flooding attacks, and, within public networks at scale, this is what would cause a greater delay. For this analysis, we have only considered a PowTime of two seconds on whisper, to be compared to the actual propagation times seen though the Telegram API and our solution based on Tor hidden services.

### 5.1. Computational Overhead

We used the web browser plugin Metamask (https://metamask.io/) and the light-client Geth (https://www.ethereum.org/cli) implementation for our decentralized application. The light-client implementation allows patients and doctors to issue transactions without needing to store a full copy of the remote healthcare blockchain. Additionally, since our nodes do not mine and validate new blocks, the computational overhead on the Metamask implementations is negligible.

### 5.2. Helthcare Data Propagation Time

**Figure 4:**
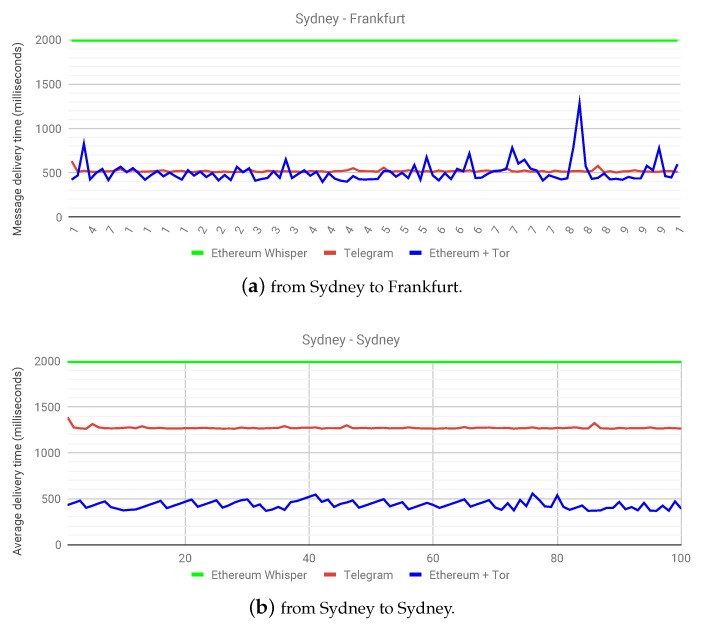
Message delivery times when delivering remote health monitoring data (**a**) from Sydney to Frankfurt and (**b**) from Sydney to Sydney.

To compare message delivery times, we set up virtual machines on the Google Compute Engine service. We connected these VMs to the Ethereum Rinkeby network and Tor. While delivering messages, we observed the time taken through a centralized solution (Telegram) and our proposed solution. As already mentioned, we have only considered two seconds of PowTime for Ethereum’s whisper (not including any actual propagation time). Figure 4a shows the message delivery times between two nodes, one in Sydney and another in Frankfurt. Not only does this highlight the potential in globalizing healthcare, it also shows that the solution proposed in this work performs comparatively to the centralized solution at a near real-time level. This experiment confirms the hypothesis of a slightly higher delivery time than the centralized solution, but a marked improvement over whisper on a public Ethereum network.

A more interesting outcome presents itself in Figure 4b, where, say, a patient from Sydney wants to transfer health monitoring data to a doctor in Sydney. Since Telegram has centralized servers, as is the case with current health monitoring systems, the latency in delivering data streams becomes clear over various geographical locations. In this case, the centralized solution clearly has a double delivery time in milliseconds, owing to the fact that the centralized service does not have servers in Australia, while our proposed solution is making use of a decentralized infrastructure already in place. Recordkeeping events over Ethereum only occur during the start and end of a data stream, and the graphs in Figure 4 illustrate the fact that the latency introduced in using Ricochet is comparable to the experience provided by centralized services. In some instances, it may perform better than centralized services with distant servers. This experiment has helped highlight not only the capabilities of our proposed solution, but also the benefits of a decentralized architecture as a whole. As for the future work, we plan to observe the effect of parameter changes in health data propagation time. We aim to benchmark our proposed solution with various setups in the number of patients, doctors, and hospitals.

### 5.3. Performance under Varying Number of Participants

Currently, Ethereum has a performance limit of handling 15 transactions per second. However, with the newer Istanbul release (https://www.coindesk.com/ethereums-istanbul-hard-fork-is-now-live), Ethereum is expected to provide a much larger throughput. Given this current limitation in mind, we have designed the framework to only issue transactions at the start and end of monitoring sessions, for the purpose of censorship-resistant recordkeeping, while the actual monitoring data are transmitted through off-chain methods. Minimising the load on the Ethereum blockchain itself allows for more healthcare providers to join the network and perform remote health monitoring.

**Figure 5:**
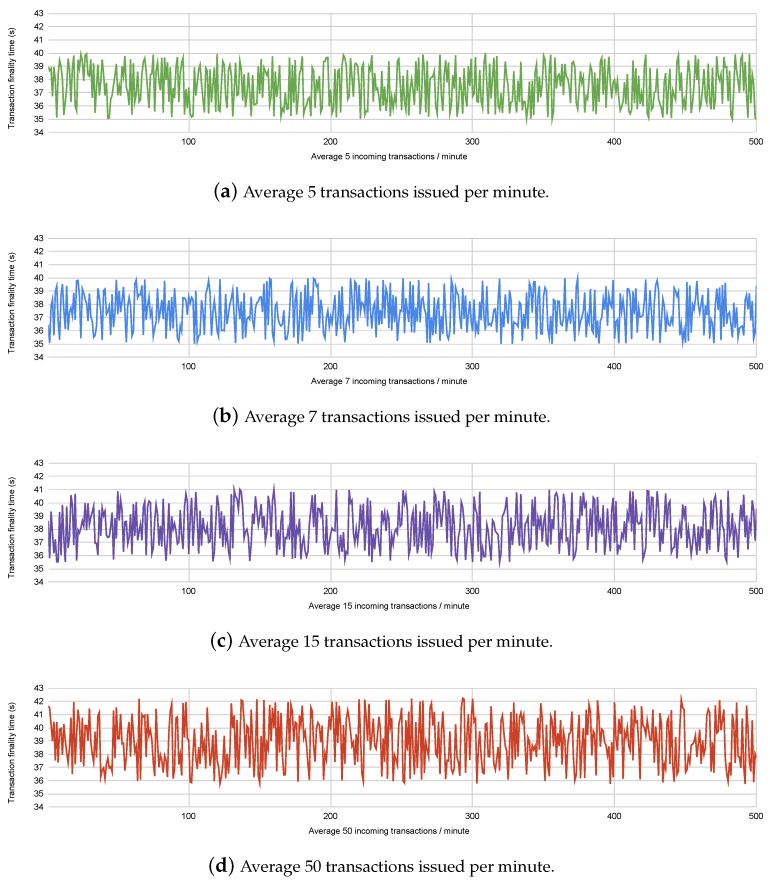
Transaction finality times for 500 transactions issued over Ethereum Rinkeby with varying incoming transaction rates.

To compare the performance of the framework with varying number of healthcare providers, we conducted experiments on Ethereum’s Rinkeby testnet, where we issued transactions with varying rates of incoming transactions. We have considered the incoming transaction rates under assumptions of the number of participating healthcare providers and patients. For example, 100 hospitals performing monthly remote health monitoring of an average of 1000 patients each would issue an average of 200,000 transactions per month, or 4.6 transactions per minute. The different incoming transaction rates considered in the experiment are shown in Figure 5. Transaction finality times indicate the time taken for the transaction to be published onto the blockchain. The Ethereum blockchain has a fixed block time of 15 seconds, which means each transaction stands a chance of being added onto the blockchain within the 2nd or 3rd block published since the time the transaction was issued. We have observed the transaction finality times for 500 transactions incoming at varying speeds, to observe the performance of the blockchain platform.

Figure 5 shows us that the multiple rates of incoming transactions have a comparable transaction finality time; however, if the rate of transactions reaches closer to 15 per second, the finality time stands to increase. The finality time for each transaction also depends upon the gas consumed by the smart contract function which resulted in the transaction being issued. We have attempted to minimise the gas consumption of the functions written in the smart contract, to reduce finality time. Table 1 shows the amount of gas consumed by the functions written in the smart contract.

## 6. Related Work

Recent related work in developing decentralized IoT RHM systems show us various techniques for handling the challenges associated with integrating blockchains into the IoT. Data transfer over blockchains can prove to be problematic in terms of the heavy latency involved; therefore, for data delivery, off-chain mechanisms are necessary. The solution described in [27] involves centralized authoritative entities that set up end-to-end data delivery channels to enhance patient security. For decentralizing both the control plane and data delivery plane within a blockchain-based solution, and on-chain/off-chain hybrid is the more evident solution. Towards a hybridization of on-chain and off-chain solutions, Enigma [28] introduced off-chain cloud-based storage and utilizes distributed-hash-tables for IoT data management. Further down into hybrid on-chain/off-chain solutions, [19] uses IPFS as a decentralized storage medium to share static data files, while maintaining records on blockchains.

A multitude of research work has been recently carried out in the healthcare space in general, involving blockchains as a medium for accountable record-keeping. Within the health insurance industry, MIStore proposes a blockchain-based medical insurance system, deployed on the Ethereum blockchain platform [29]. It uses the immutability of blockchain storage to maintain insurance records. Additionally, Pokitdok [30] aims to facilitate insurance claim resolution in healthcare industrial verticals.

Another popular use-case for blockchains in healthcare has been in electronic medical record-keeping [8]. Such solutions perform creation, storage and management of patients’ health records over a consortium blockchain. Some solutions aim to share patient-centric data among different healthcare stakeholders while adhering to GDPR regulations [31]. Guardtime [32] is regularly cited as the most successful medical record-keeping project, and it secures over a million patient records in Estonia. To secure medical records collected at the patients’ end through mobile devices, the authors in [33] propose a blockchain-based data management system, with practical swarm optimization for root exploit detection and feature optimization.

Blockchains and the IoT have also been the subject of research in the area of remote health monitoring. Remote health monitoring entails the collection of biomedical data through wearable sensors to monitor the physical condition of remotely located patients. Recent work has gone into storing, managing and sharing remotely-collected patient data through blockchain-based systems [34,35,36]. In [37], authors propose a solution built on the Ethereum platform with smart contracts to support real-time monitoring with automated interventions and preemptive measures. The solution may work best in a smaller consortium, but the inherent latency in Ethereum’s transaction processing over public deployments may hinder the efficacy of the solution. Blockchain-based solutions are also being worked on for medical identity management. Along with developing solutions, studies on patient privacy are also ongoing in blockchain-based identity management [38].

Liang et al. [39] propose a solution implemented on the Hyperledger platform, which aims to share patient monitoring data among healthcare stakeholders in an online healthcare environment. Similarly, SMEAD [40] is a mobile-enabled assisting device with a blockchain client for monitoring diabetic patients. Another solution proposes using blockchain-based patient-centric agents for end-to-end security [41]. The idea here is to use automated blockchain-based agents as mediators for automated healthcare monitoring. Fernandez et al. [42] propose cryptocurrency for incentivizing users to crowdsource medical data for health monitoring as well as medical research. To secure medical records collected at the patients’ end through mobile devices, the authors in [33] propose a blockchain-based data management system, with practical swarm optimization for root exploit detection and feature optimization. Dwivedi et al. [43] leverage the security features of blockchains to develop a health monitoring system; however, data transactions all occur over the blockchain, which is not a scalable solution. In comparison, the decentralized health monitoring framework outlined in this work aims to leverage blockchain-based security for identity management and record-keeping, along with off-chain data transfer mechanisms for circumnavigating blockchain transaction delays and improving scalability.

## 7. Conclusions

Today, a blockchain-IoT convergence is seen as a potential step forwards towards democratic and decentralized healthcare systems. However, despite some valuable solutions recently proposed in the literature, blockchain technology still remains nascent, though it is expected to yield several interesting outcomes for the future. In this paper, we proposed a private-by-design, blockchain-based solution for remote health monitoring. We built the solution on the Ethereum blockchain platform, and we used Tor hidden services for off-chain data delivery. We demonstrated the proposed solution through a full-fledged implementation and made important observations by conducting a performance analysis. Our results showed that, in terms of message delivery times, the solution performed on par with centralized solutions when the centralized servers were halfway in between the patient and the doctor. Moreover, our analysis showed that, in scenarios where the centralized servers were distant, our solution had an improved performance due to its non-reliance on centralized servers. To conclude, by designing and implementing an on-chain and off-chain, we have taken steps towards achieving a private-by-design solution for remotely monitoring patients’ health.

## Figures and Tables

**Table 1:** Gas usage and transaction fees for executing functions in the remote health monitoring (RHM) smart contract.

Functions	Invoked By	When it is Invoked	Gas Usage	ETH Price
REQUESTmathsizesmallDATAmathsizesmall()	Healthcare provider	Preamble to stream session	746,108	0.000746108
STARTOFmathsizesmallSTREAMmathsizesmall()	Patient’s IoT GW	Once per session	27,978	0.000027978
ENDOFmathsizesmallSTREAMmathsizesmall()	Patient’s IoT GW	Once per session	63,251	0.000063251
